# Application of *Ng*Ago-mediated genome editing in *Mycobacterium smegmatis*

**DOI:** 10.1128/jb.00214-25

**Published:** 2025-08-21

**Authors:** Li Zhao, Shi-Qi Yang, Yu-Wei Feng, Bang-Ce Ye, Di You

**Affiliations:** 1 Lab of Biosystems and Microanalysis, State Key Laboratory of Bioreactor Engineering, East China University of Science and Technology, Shanghai, China; 2 Institute of Engineering Biology and Health, Collaborative Innovation Center of Yangtze River Delta Region Green Pharmaceuticals, College of Pharmaceutical Sciences, Zhejiang University of Technology, Hangzhou, Zhejiang, China; University of Notre Dame, Notre Dame, Indiana, USA

**Keywords:** *Ng*Ago-F, gene editing, glnR, ltmA

## Abstract

*Mycobacterium smegmatis* is nonpathogenic and fast-growing and is usually used as a model species of *Mycobacterium*. Studying basic metabolic mechanisms is crucial for accelerating mycobacterial research. Although several tools for genome editing in *Mycobacterium smegmatis* MC ^(2)^ 155 (*M. smegmatis*) can be used, plasmids are difficult to construct, and the knockout efficiency is still low. Here, the *Ng*Ago system was utilized to edit the genome of the Gram-positive bacterium *M. smegmatis*, which has a high guanine-cytosine (GC) content. A shuttle plasmid containing the hsp60 promoter to drive *Ng*Ago expression was designed. PCR-mediated screening and qRT‒PCR confirmed that the *glnR* gene (KEGG: *MSMEG_5784*) and *ltmA* gene (KEGG: *MSMEG_6479*) were successfully knocked out by the *Ng*Ago-F system. The editing efficiency reached 80%, and the time requirement was reduced to 8 days. The optimized *Ng*Ago system establishes an efficient genome-editing platform for high-GC mycobacteria, advancing functional genomics research on *M. smegmatis* and potentially enabling precise interrogation of virulence mechanisms in pathogens, such as *Mycobacterium tuberculosis*.

**IMPORTANCE:**

In this work, we demonstrated that the *Ng*Ago system could be used to edit the genome of *Mycobacterium smegmatis* and has several advantages: easy plasmid construction, high editing efficiency, and short time requirements. These findings provide a powerful tool for elucidating the basic metabolic mechanisms of *M. smegmatis* and potentially those of other mycobacterial species.

## INTRODUCTION

Tuberculosis (TB), caused by *Mycobacterium tuberculosis*, is a highly lethal disease that is transmitted primarily through the respiratory tract. According to the “Global Tuberculosis Report 2024,” released in 2024, the global death toll from tuberculosis reached 1.25 million, replacing COVID-19 as the world’s leading cause of death from a single infectious agent. Research on the mechanism of pathogenicity in *M. tuberculosis* is currently a key focus in tuberculosis prevention and control efforts (1). However, current limitations in mycobacterial genetic tools, particularly low editing efficiency and technical constraints, pose significant barriers to studying TB pathogenesis and developing effective interventions. To address these methodological challenges, the nonpathogenic model organism *M. smegmatis* was selected for this study due to its unique experimental advantages. Its fast growth cycle, genetic accessibility, and safe handling characteristics make *M. smegmatis* exceptionally suitable for developing and optimizing improved genetic manipulation systems (2). Establishing efficient and universal genetic manipulation platforms using *M. smegmatis* is expected to substantially accelerate investigations into *M. tuberculosis* pathogenesis-related gene functions and provide critical technical support for the development of novel anti-TB strategies, complementing direct studies in *M. tuberculosis*.

Traditional genome editing methods for *M. smegmatis* rely on allele exchange, homologous recombination, and nonhomologous end joining (NHEJ), but the knockout efficiency is very low (10^−5^‒10^−6^) (3). With the development of CRISPR technology, CRISPR/dCas9 has been shown to reduce gene expression in *M. smegmatis* (4). The CRISPR-Cas12a-assisted homologous recombination system was subsequently used for genome editing in *M. smegmatis*, and the efficiency of introducing site-specific point mutations reached 80%, whereas the deletion/insertion efficiency of double-stranded DNA recombination was 37%–75% (5). Recent studies have shown that the CRISPR-FnCpf1-assisted NHEJ system can repair double-strand breaks (DSBs) in *M. smegmatis* without requiring recombinant proteins or homologous DNA templates, enabling single-gene knockout with a total time requirement of 7 days (6). However, although this CRISPR-mediated knockout system provides a tool for rapid and efficient mycobacterial genome modification, challenges associated with its use exist, such as the complexity of the construction of plasmids harboring sgRNA, varying efficiencies of different sgRNAs, off-target effects, and the large size of cas protein-containing plasmids, leading to difficulties in vector construction and transformation. These challenges limit the application of this system in actinomycetes, in which plasmid transformation is difficult. The development of a simple and efficient tool for rapid genome editing could accelerate functional genomic investigations of *M. smegmatis* and be beneficial for exploring the mechanism of *M. tuberculosis* pathogenesis.

The Argonaute (Ago) protein is a programmable nucleic acid enzyme found in both eukaryotes and prokaryotes. Studies have shown that while prokaryotic Ago protein (pAgo) and eukaryotic Ago protein (eAgo) share high structural homology, eAgo proteins can cleave only RNA targets, whereas many pAgo proteins can cleave DNA targets (7). In prokaryotes, Agos are divided into long pAgos and short pAgos, among which long pAgos consist of four domains, namely, the N-terminal, PIWI–Argonaute–Zwille (PAZ), middle (MID), and PIWI domains, as well as the L1 and L2 linkers, whereas short pAgos consist of only the MID and PIWI domains (7–9). Many pAgos, such as *Tt*Ago (10), *Mj*Ago (11), and *Pf*Ago (12), can function only at temperatures above 65°C with gDNA as the guide. However, *Ng*Ago was found to function at 30°C or 37°C, and this temperature is suitable for the growth of most organisms (8, 13). In 2016, Gao et al. (13) discovered that *Ng*Ago functions as a DNA-guided endonuclease and is applicable for genome editing in human cells. However, subsequent studies by other research teams were unable to confirm that *Ng*Ago possesses gene-editing capabilities. In the same year, Qi et al. reported that gDNA/*Ng*Ago can bind to target genes to block transcription in zebrafish, a eukaryotic organism, but no gene-editing activity of *Ng*Ago was observed (14). In 2019, Fu et al. confirmed that *Ng*Ago physically interacts with RecA to mediate homologous recombination in bacteria (8). In 2021, Lee et al. confirmed that *Ng*Ago has guided DNA nicking activity and further demonstrated that *Ng*Ago performs targeting and gene editing in *E. coli* (15). In 2022, Xing et al. reported that *Ng*Ago had DNA cleavage activity similar to that of Cas9 (16). Although *Ng*Ago cannot knock out genes in eukaryotic organisms, it can function in prokaryotes, such as *Pasteurella multocida* and *Escherichia coli*, through RecA-mediated recombination (8). The prokaryotic-specific activity of *Ng*Ago arises from its dependence on RecA, compatibility with bacterial DNA repair pathways, and evolutionary optimization for prokaryotic physiology. In contrast, eukaryotic barriers—including incompatible repair mechanisms and host immune responses—preclude editing. In addition, researchers have analyzed the truncated domains of the *Ng*Ago protein and reported that the last 717 bp of the PIWI domain, namely, *Ng*Ago-F, is the primary domain that mediates bacterial genome editing (8). *Ng*Ago-F can interact with the intracellular homologous recombination enzyme RecA, increasing the RecA-mediated homologous DNA strand exchange capability and thereby increasing recombination efficiency, and the knockout efficiency can reach 80%–100% (8). In this study, the *Ng*Ago-F system was developed for genome editing in *M. smegmatis*. The constitutive promoter of a heat shock protein, Hsp60 (6), was used to drive *Ng*Ago-F expression in pKC1139 (17). GlnR, a global transcriptional regulator of nitrogen metabolism, and LtmA, a receptor for c-di-GMP, were chosen as the targets, and the upstream and downstream homology arm sequences were then inserted into pKC1139. After transformation into *M. smegmatis*, qRT-PCR and sequencing were performed to identify whether the *glnR* or *ltmA* genes were successfully knocked out. To further reduce the plasmid size, we compared the effects of homology arm length on the knockout efficiency of the *glnR* gene in *M. smegmatis*. The 200 bp homologous arm sequences were sufficient to successfully knock out the *glnR* gene. In summary, the *Ng*Ago-F-based system provides a quick, efficient, and easy-to-use gene editing tool for use in *M. smegmatis*, with significant potential for application in tuberculosis prevention and treatment research.

## RESULTS

### Plasmid construction

**Fig 1:**
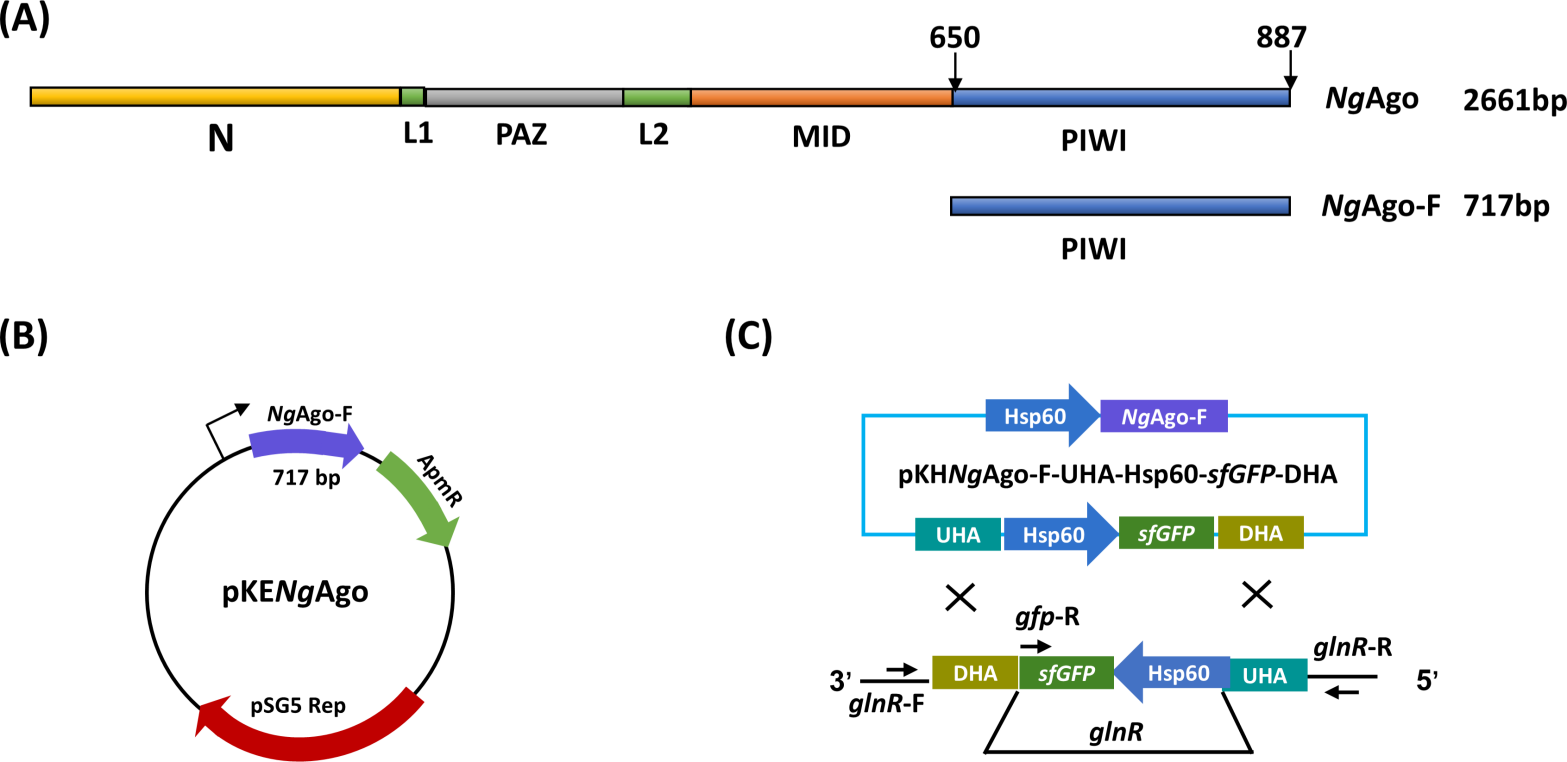
Construction of knockout-related plasmids. (**A**) Domain organization of *Ng*Ago and *Ng*Ago-F. (**B**) Composition of the *Ng*Ago-F-mediated plasmids. A Flag*-Ng*Ago-F gene with an *Hsp*60 promoter was introduced into the plasmid pKC1139 to generate the plasmid pKH*Ng*Ago-F. (**C**) Scheme of *glnR* gene knockout in *M. smegmatis* via the *Ng*Ago-F system.

*Ng*Ago-F is the PIWI domain of *Ng*Ago, which is approximately 717 bp in length and mediates bacterial genome editing (Fig. 1A). We codon-optimized the sequence of the *Ng*Ago-F gene to make *Ng*Ago-F much easier to express and function in *M. smegmatis*. Using homologous recombination, we successfully inserted Flag-*Ng*Ago-F driven by the *Hsp*60 promoter into the shuttle plasmid pKC1139, which contains a temperature-sensitive pSG5 replication system and a cassette encoding the aac(3)-Ivan gene for apramycin resistance that can be subsequently lost through temperature treatment (18). Through this method, the pKC1139-Hsp60-Flag-*Ng*Ago-F (pKH*Ng*Ago-F) plasmid was constructed (Fig. 1B). Then, upstream and downstream sequences, with superfolder green fluorescent protein (*sfGFP*) between them, were introduced into the pKH*Ng*Ago-F plasmid. A schematic diagram of the constructed knockout plasmid and the knockout principle is shown in Fig. 1C.

### Solubility verification analysis of *Ng*Ago-F

**Fig 2:**
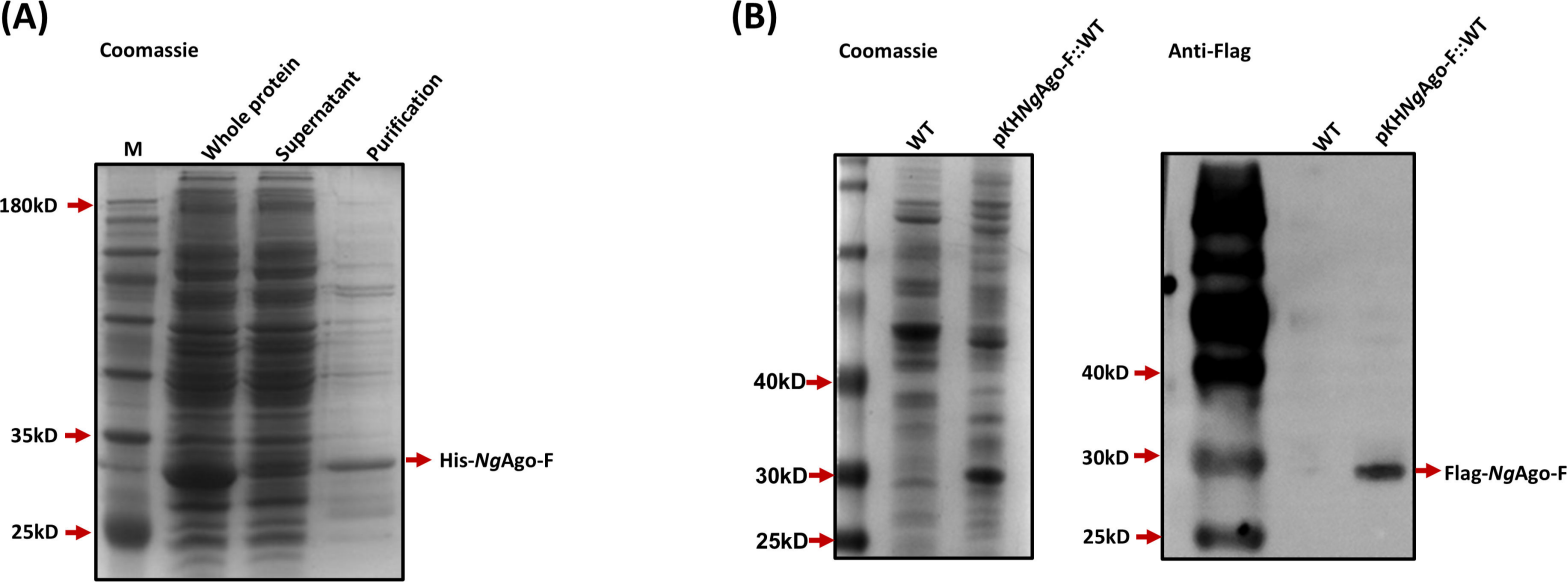
*Ng*Ago-F is soluble in *E. coli* and *M. smegmatis*. (**A**) His-*Ng*Ago-F protein expression and purification in BL21(DE3) cells. The His-*Ng*Ago-F protein was separated by 15% SDS‒PAGE and stained with Coomassie Brilliant Blue R-250. (**B**) The Flag-*Ng*Ago-F protein was expressed in *M. smegmatis* and identified via western blotting with an anti-Flag antibody. Left: Coomassie blue staining of supernatant protein from WT and pKH*Ng*Ago-F::WT. Right: Western blotting measurement of *Ng*Ago-F in the supernatants of WT and pKH*Ng*Ago-F::WT.

The solubility of the *Ng*Ago-F protein is critical to its function. To verify the solubility of *Ng*Ago-F, we first constructed the pET28a-His-*Ng*Ago-F plasmid via the homologous recombination method. The plasmid was subsequently transformed into BL21(DE3) cells. His-*Ng*Ago-F, a 31.2 kDa fusion protein, was successfully expressed and purified (Fig. 2A), demonstrating that *N*gAgo-F is soluble in *E. coli*. To further confirm whether the *N*gAgo-F protein is soluble in *M. smegmatis*, we transferred pKH*Ng*Ago-F into the wild-type (WT) strain and obtained the strain pKH*N*gAgo-F::WT. Western blotting was performed using an anti-Flag antibody. The *N*gAgo-F protein was detected in the supernatant, confirming that the *N*gAgo-F protein is also soluble in *M. smegmatis* (Fig. 2B). These results indicate that *N*gAgo-F can achieve genome editing in *M. smegmatis*.

### Deletion of *glnR* in *M. smegmatis*

**Fig 3:**
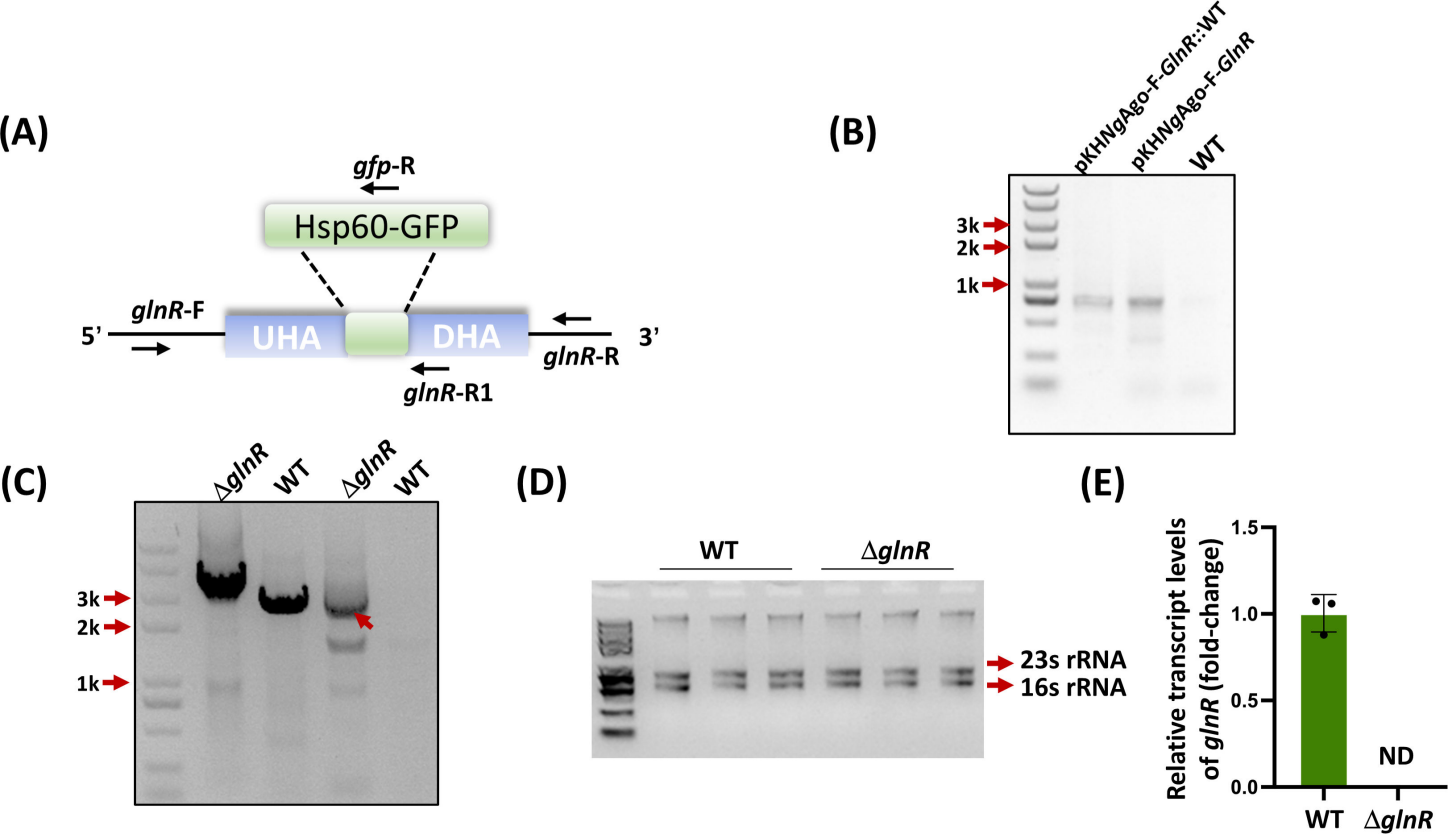
*glnR* deletion in *M. smegmatis*. (**A**) *glnR* in genomic DNA was replaced with *Hsp*60-*GFP*. (**B**) Plasmid pKH*Ng*Ago-F-*GlnR* was transformed into WT through PCR identification. Plasmid pKH*Ng*Ago-F-*GlnR* and WT strain represent positive and negative controls, respectively. (**C**) *glnR* knockout identification of the strains via PCR amplification using the primers *glnR*-F/R (left two lines) and *glnR*-F/*gfp*-R (right two lines), with Δ*glnR* and WT as the templates. (**D**) Agarose gel electrophoresis of total RNA (with genomic DNA) extracted from WT and Δ*glnR*, and the resulting RNAs were not degraded. (**E**) Relative expression of *glnR* in the WT and Δ*glnR* strains. ND: not detected. The error bars indicate the SDs of three independent biological experiments, and the mean values were calculated on the basis of these replicates.

To determine whether the *Ng*Ago-F tool can achieve genome deletion in *M. smegmatis*, we first constructed a plasmid for *glnR* knockout. To ensure high cleavage efficiency, we recombined 800 bp upstream and 800 bp downstream homology arms into the pKH*Ng*Ago-F plasmid and obtained the plasmid pKH*Ng*Ago-F-*GlnR*. After the plasmid was transformed into *M. smegmatis* through electroporation for 3‒4 days, individual colonies were transferred to liquid LB media for further cultivation, and PCR was performed to confirm that the plasmids were successfully transferred into *M. smegmatis* (Fig. 3B). After three passages, PCR was performed, and the products were sequenced to further confirm that the *glnR* gene was indeed knocked out in strains with successfully transferred plasmids (Fig. 3A and C). Additionally, we extracted RNA from the WT and Δ*glnR* strains and conducted qRT‒PCR analysis. Our results revealed that in the Δ*glnR* mutant, the expression of *glnR* was absent, which indicated that *glnR* was knocked out successfully and that the *Ng*Ago-F system can be used for genome editing in *M. smegmatis* (Fig. 3D and E).

### Deletion of *ltmA* in *M. smegmatis*

**Fig 4:**
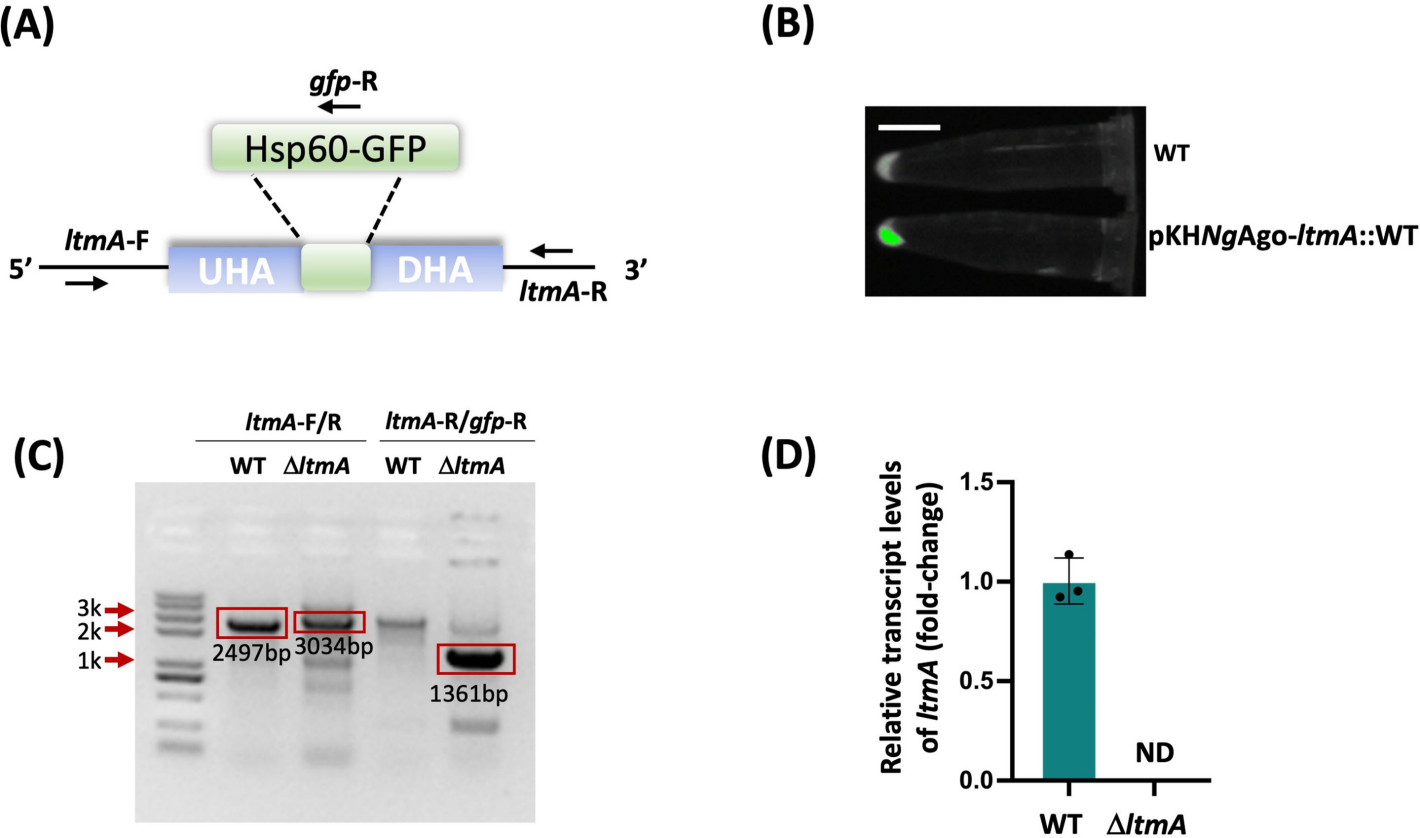
*ltmA* deletion in *M. smegmatis***.** (**A**) *ltmA* in the genomic DNA was replaced with *Hsp*60-*GFP*. (**B**) The fluorescence signal suggested that the plasmid with *GFP* was successfully transformed into *M. smegmatis.* The signal was captured via a ChemiScope 6100 chemiluminescence imaging system. Scale bars, 1 cm. (**C**) PCR-mediated identification of the recombinant strains via the *ltmA*-F/R and *ltmA*-F/*gfp*-R primers. The 2,497 bp band is the length amplified via the primers *ltmA*-F/R with the WT as the template. The 3,034 bp band is the length amplified via the primers *ltmA*-F/R with the Δ*ltmA* mutant template. The 1,361 bp band is the length amplified via the primers *ltmA*-R/*gfp*-R with the Δ*ltmA* mutant. (**D**) Relative expression of *ltmA* in the WT and Δ*ltmA* strains. ND: not detected. The error bars indicate the SDs of three independent biological experiments, and the mean values were calculated on the basis of these replicates.

To test whether *Ng*Ago-F-based gene knockout in *M. smegmatis* could be applied to genes other than *glnR*, we applied this approach to manipulate another gene, *ltmA*. We recombined the 800 bp upstream and downstream homology arms of the *ltmA* gene and *sfGFP* fragment into the pKH*Ng*Ago-F vector, obtaining the plasmid pKH*Ng*Ago-F-*ltmA*. The plasmid was then transferred into *M. smegmatis*, and single colonies were selected for cultivation. Through fluorescence signals, we identified the strains that were successfully transformed with the plasmid pKH*Ng*Ago-F-*ltmA* (Fig. 4B). After three passages, we confirmed the deletion of *ltmA* via PCR amplification with the primers *ltmA*-F/R and *ltmA*-R/*gfp*-R and obtained the Δ*ltmA* mutant (Fig. 4A and C). To further substantiate the knockout results, RNA was extracted, and qRT-PCR analysis revealed that the expression of *ltmA* was eliminated in the Δ*ltmA* mutant (Fig. 4D). These results further support that the *Ng*Ago-F system can be used for genome editing in *M. smegmatis.*

### Influence of homology arm length on *Ng*Ago-F-assisted gene editing efficiency

**Fig 5:**
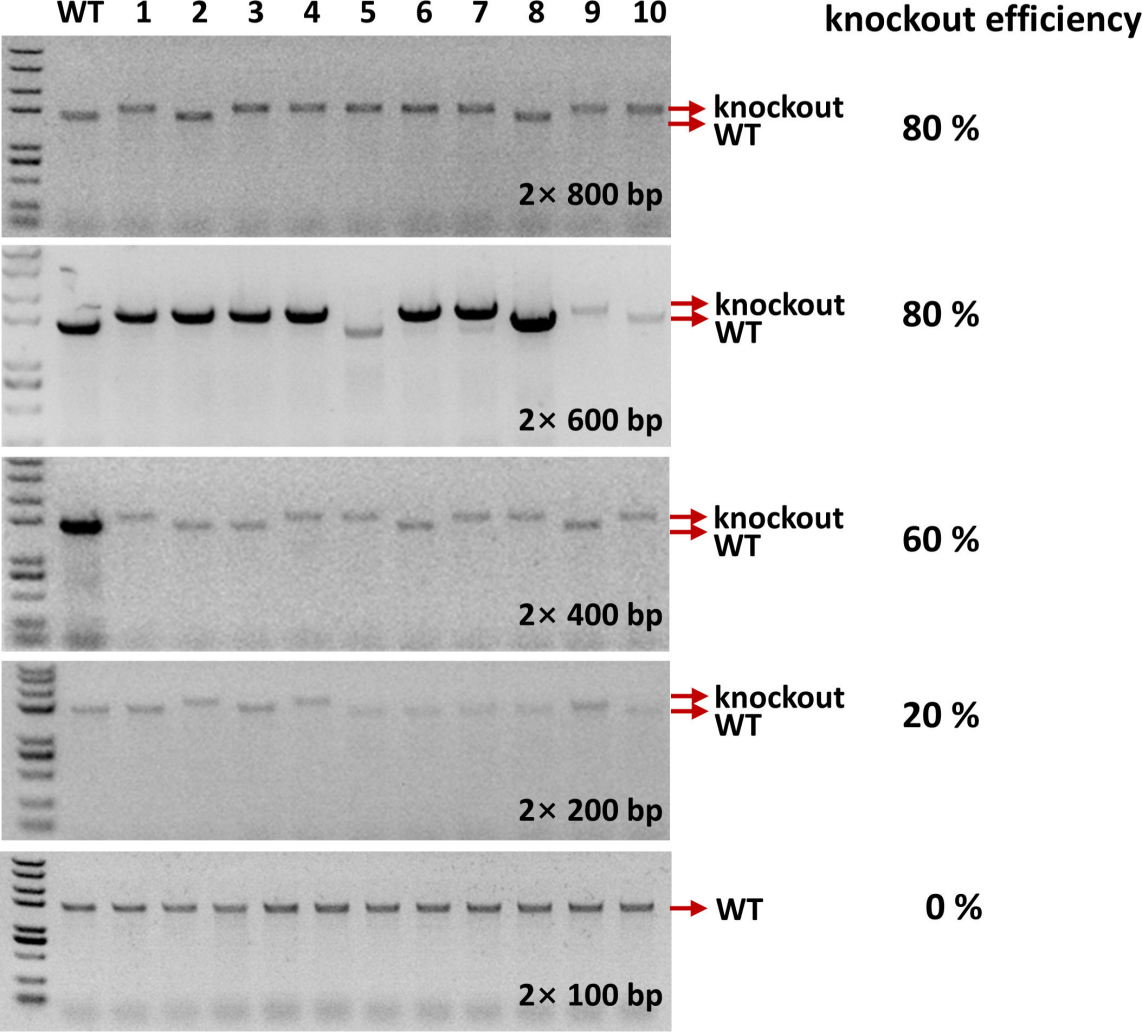
Impact of arm length on the *Ng*Ago-F-assisted gene editing efficiency**.** Comparison of the knockout efficiency of the *glnR* mutant in *M. smegmatis* on the basis of *Ng*Ago-F with different lengths of the homology arm. Ten clones were used to calculate the knockout efficiency with the primers *glnR*-F/R1.

Plasmid transformation is a necessary step for gene editing. The larger the plasmid is, the lower the transformation efficiency. For some difficult-to-transform strains, a small plasmid is considered essential for successful transformation. To further screen for small plasmids for *Ng*Ago-F-mediated gene editing in *M. smegmatis*, we explored the impact of homology arm length on *Ng*Ago-F cleavage activity in *M. smegmatis,* using *glnR* as the target. We constructed *glnR* gene knockout plasmids with homology arm lengths of 2 × 100 bp, 2 × 200 bp*,* 2 × 400 bp, 2 × 600 bp, and 2 × 800 bp, and separately transformed them into *M. smegmatis* to calculate the gene knockout efficiency (Fig. 5). The results indicated that the construction with a 2 *×* 100 bp homology arm length could not completely knock out the gene; a 20% knockout efficiency was achieved with a 2 × 200 bp homology arm length; a 60% knockout efficiency was achieved with a 2 × 400 bp homology arm length; an 80% knockout efficiency was achieved with a 2 × 600 bp homology arm length; and an 80% knockout efficiency was achieved with a 2 × 800 bp homology arm length (Fig. 5). Through comparison, we found a 600 bp homology arm length is sufficient to achieve efficient gene knockout, and that the minimum length of the knockout plasmid could be reduced to 200 bp. Reducing the length of the plasmid increased the likelihood of successful plasmid transformation into mycobacteria, leading to successful knockout of the target gene.

## DISCUSSION

To our knowledge, the *Ng*Ago-F system developed for *M. smegmatis* transformation in this study is easy to use and has minimal restrictions for the recognition of sequences without a PAM site. The adaptability of *Ng*Ago-F to mycobacterial genomic constraints highlights its potential to accelerate functional studies of essential metabolic pathways. For example, *glnR* knockout strains could elucidate the role of nitrogen assimilation in mycobacterial survival, whereas *ltmA* deletion may reveal lipid homeostasis mechanisms critical for pathogenicity in related species. The enhanced efficiency and reduced timeline address a critical bottleneck in mycobacterial genetics, enabling rapid validation of gene‒phenotype relationships. Moreover, the plasmid simplicity and GC-rich targeting flexibility of the *Ng*Ago platform suggest broader applicability to other Actinobacteria, which are notoriously recalcitrant to genetic manipulation. By streamlining genome editing, this approach may expedite the identification of therapeutic targets or resistance mechanisms in pathogenic mycobacteria, ultimately supporting the development of novel antimicrobial strategies.

## MATERIALS AND METHODS

### Strains, plasmids, and culture conditions

*E. coli* DH5α served as the host for plasmid construction and was used to assess NgAgo-F-assisted gene editing efficiency. *E. coli* BL21(DE3) was used for protein expression and purification. Additionally, *M. smegmatis* MC^2^ 155 served as the host for evaluating NgAgo-F-assisted gene editing. Unless otherwise stated, *E. coli* DH5α, BL21(DE3), and *M. smegmatis* were grown in LB media at 37°C and 220 rpm, with appropriate antibiotics added to the broth as needed.

### Plasmid construction

The *Ng*Ago-F gene was synthesized by Sangon Biotech Co., Ltd. (Shanghai, China), with codon optimization. To construct the pKH*Ng*Ago-F plasmid, the restriction enzymes *EcoR* I and *EcoR* V were used to obtain the pKC1139 vector. The Flag-*Ng*Ago-F fragment and *Hsp*60 promoter were amplified via the primers Flag-*Ng*Ago-F/R and *Hsp*60-F/F1/R. These two fragments have 25 bp overlapping sequences, and the two fragments were subsequently fused through PCR amplification. The fused fragment was connected to the pKC1139 vector via the pEASY-Basic Seamless Cloning and Assembly Kit (Transgene, Beijing, China). The upstream and downstream homology arms of the *glnR* gene and *ltmA* gene were amplified using *M. smegmatis* genomic DNA as the template. These homology arms were separately cloned and inserted into the pKH*Ng*Ago-F vector, generating the plasmids pKH*Ng*Ago-F-*glnR* and pKH*Ng*Ago-F-*ltmA*. Finally, pKH*Ng*Ago-F-*glnR* and pKH*Ng*Ago-F-*ltmA* were transformed into *M. smegmatis* for further applications.

### Preparation of competent *M. smegmatis* cells and electroporation methods

*M. smegmatis* was cultured in LB liquid media supplemented with 0.05% Tween 80 at 37°C for 2 days. Subsequently, 500 µL of the culture was added to 100 mL of LB liquid media containing 0.05% Tween 80 in a 500-mL shake flask and incubated at 37°C with shaking at 220 rpm for approximately 36 h. When the OD_600_ reached 0.6, the cells were immediately placed on ice and incubated for 30 min. The cells were harvested and collected by centrifugation at 6,000*×g* at 4°C for 10 min. After the supernatants were removed, the cells were washed once with cold water and washed twice with cold 10% glycerol. Finally, the cells were resuspended in 500 µL of 10% glycerol, and 200 µL aliquots were divided into 1.5 mL sterile centrifuge tubes.

For electroporation, 20 µL of plasmid DNA was added to 200 µL of competent cells, and the mixture was transferred into precooled 2-mm electroporation cuvettes. Electroporation was performed at 3 kV for 4 ms once or twice via a MicroPulser (Bio-Rad, 411BR7769). Immediately, 900 µL of LB medium was added to the competent cells, and the mixture was incubated with shaking at 30°C and 220 rpm for approximately 3 h. Following recovery, the cells were centrifuged, and the cells in the final 200 µL were resuspended and plated on LB agar plates containing 50 µg/µL apramycin. The plates were incubated for 3‒4 days. To ensure transformation efficiency, competent cells should be prepared and used immediately.

### Protein expression and purification

The primers 28a-*Ng*Ago-F-F/R were used to amplify the *Ng*Ago-F fragment, and the primers pET28a-F/R were used to amplify the pET28a vector fragment. The two fragments had 25 bp overlapping sequences, and the overlapping sequences were designed with the primers pET28a-F/R. In the pEASY-Basic Seamless Cloning and Assembly Kit, a basic mixture was used. The assembly system contained 6 µL of Basic Mix, x µL of *Ng*Ago-F fragment, and (6−x) µL of pET28a vector, and the mixture was incubated at 50°C in a water bath for 30 min. The molar ratio of the *Ng*Ago-F fragment to the pET28a vector was 3:1‒5:1. The mixture was subsequently transformed into competent cells. T7 and T7 Ter primers were used to identify the correct clones. The recombinant plasmid pET28a-*Ng*Ago-F was transformed into *E. coli* BL21(DE3) competent cells for protein expression. The transformed cells were initially cultured in LB liquid media at 37°C overnight. Then, 5 mL was added to 100 mL of LB liquid media in a 500 mL shake flask and cultured at 37°C with shaking at 220 rpm for approximately 2‒3 h. When the OD_600_ reached 0.6, His-*Ng*Ago-F was induced by adding 0.5 mM IPTG, followed by incubation at 18°C for 14‒16 h. The cells were collected by centrifugation at 6,000*×g* for 10 min and washed once with 1× PBS. After being resuspended in 35 mL of 1× PBS, the cells were lysed via sonication, and the supernatants were collected via centrifugation at 9,000*×g* for 20 min at 4°C. Protein purification was performed according to previously established protocols (19). The purified protein was analyzed by 15% SDS‒PAGE (Epizyme Biotech, Shanghai, China).

### Western blotting

pKH*Ng*Ago-F::*M. smegmatis* and *M. smegmatis* were cultured in LB liquid media supplemented with 0.05% Tween 80 at 30°C for 2 days. The cells were collected by centrifugation at 6,000*×g* for 20 min and washed once with 1× PBS. The cell pellet was resuspended in 300 µL of 1× PBS and lysed by sonication, and the supernatants were collected by centrifugation at 9,000*×g* for 20 min at 4°C. The supernatants of pKH*Ng*Ago-F::*M. smegmatis* and *M. smegmatis* were mixed with 6× protein loading buffer and run on SDS‒PAGE gels. Next, the proteins on the SDS‒PAGE gels were transferred to a PVDF membrane (Merck, Millipore) at 300 V and 380 mA for 90 min. After being washed twice with TBST buffer, which consisted of 20 mM Tris-HCl, pH 7.6, 150 mM NaCl, and 0.1% (v/v) Tween 20, the PVDF membrane was incubated with 0.5% BSA blocking buffer at 4°C overnight. Two microliters of anti-Flag antibody (Transgene, Beijing, China) was added to the BSA blocking buffer and incubated at 25°C for 1.5 h. Then, the PVDF membrane was washed three times with TBST buffer, and 2 µL ProteinFind Goat Anti-Mouse IgG (H + L) (Transgene, Beijing, China) was added to 10 mL of TBST buffer. After incubation for approximately 1 h, the PVDF membrane was washed three times with TBST, and an enhanced chemiluminescence (ECL) system (CTB, USA) was used to detect the signal.

### qRT‒PCR

WT, Δ*glnR*, and Δ*ltmA* were cultured in LB media supplemented with 0.05% Tween 80 at 37°C for 2 days. Total RNA was extracted from WT, Δ*glnR,* and Δ*ltmA* strains with an RNAprep Pure Cell/Bacteria Kit (Tiangen Biotech, Beijing, China) according to the manufacturer’s instructions. cDNA was subsequently synthesized via TransScript Uni All-in-One First-Strand cDNA Synthesis SuperMix (Transgene, Beijing, China) for qRT‒PCR. A 96-well reaction was performed with a Bio-Rad CFX96 real-time PCR detection system (Bio-Rad, Hercules, CA, USA) using TB Green Premix Ex Taq (TaKaRa Biotechnology, Beijing, China). All PCR mixtures were prepared in a volume of 20 µL in triplicate and contained 10 µL of SYBR Green PCR master mix, 0.4 µM forward primer, and 0.4 µM reverse primer. The PCR conditions were as follows: 95°C for 5 min, followed by 40 cycles of 95°C for 30 s, 55°C for 20 s, 72°C for 15 s, and 72°C for 10 min. The expression levels of *glnR* and *ltmA* were normalized to that of the housekeeping gene *sigA* (*MSMEG_2758*).

## SUPPLEMENTAL MATERIAL

Supplemental tables and figure jb.00214-25-s0001.docx Tables S1 and S2, and Fig. S1 to S3.

ASM does not own the copyrights to Supplemental Material that may be linked to, or accessed through, an article. The authors have granted ASM a non-exclusive, world-wide license to publish the Supplemental Material files. Please contact the corresponding author directly for reuse.
