# Supplementary material for: Application of NgAgo-mediated genome editing in Mycobacterium smegmatis
Source: J Bacteriol. 2025 Aug 21;207(9):e00214-25. doi: 10.1128/jb.00214-25 (PMC12445092; doi:10.1128/jb.00214-25)
Supplement: Supplemental tables and figure — Tables S1 and S2, and Fig. S1 to S3. [file jb.00214-25-s0001.docx]

Supplementary Material:

**Application of NgAgo Mediated Genome Editing in Mycobacterium smegmatis**

Li Zhao^a#^, Shi-qi Yang^a#^, Yu-Wei Feng^a^, Bang-Ce Ye^a,b^, Di You^a*^

^a^*Lab of Biosystems and Microanalysis, State Key Laboratory of Bioreactor Engineering, East China University of Science and Technology, Shanghai 200237, China*

^b^*Institute of Engineering Biology and Health, Collaborative Innovation Center of Yangtze River Delta Region Green Pharmaceuticals, College of Pharmaceutical Sciences, Zhejiang University of Technology, Hangzhou 310014, Zhejiang, China*

^#^Li Zhao and Shi-Qi Yang contributed equally to this article.

**Corresponding authors**

Di You

Associate Professor, Lab of Biosystems and Microanalysis,

State Key Laboratory of Bioreactor Engineering,

East China University of Science and Technology, Shanghai 200237, China

Tel/Fax: 0086-21-64253832

Email: [030111115@mail.ecust.edu.cn](mailto:030111115@mail.ecust.edu.cn)

**Table S1 Strains and plasmids used in this work**

| **Strains or Plasmids** | **Characters** | **Sources** |
| --- | --- | --- |
| **Strains** |  |  |
| *E. coli* DH5α | *E. coli* DH5α F‐Ø80d lacZΔM (lacZ-YA‐argF) U169 deoR | Transgen Biotech |
| *E. coli* BL21(DE3) | F'ompTr‐Bm‐B (DE3) | Transgen Biotech |
| *E. coli* BL21(DE3)::pET28a-*Ng*Ago-F | The strain to expression of *Ng*Ago-F | This study |
| *M. smegmatis* |  | Lab-preserved |
| pKH*Ng*Ago-F::*M. smegmatis* | The strain to identify the dissolubility of *Ng*Ago-F | This study |
| pKH*Ng*Ago-F-*glnR*-*sfGFP*::*M. smegmatis* | The strain to knock out *glnR* | This study |
| pKH*Ng*Ago-F-*ltmA*-*sfGFP*::*M. smegmatis* | The strain to knock out *ltmA* | This study |
| pKH*Ng*Ago-F-*glnR*-*sfGFP*-100 bp::*M. smegmatis* | The strain to knock out *glnR* | This study |
| pKH*Ng*Ago-F-*glnR*-*sfGFP*-200 bp::*M. smegmatis* | The strain to knock out *glnR* | This study |
| pKH*Ng*Ago-F-*glnR*-*sfGFP*-400 bp::*M. smegmatis* | The strain to knock out *glnR* | This study |
| pKH*Ng*Ago-F-*glnR*-*sfGFP*-600 bp::*M. smegmatis* | The strain to knock out *glnR* | This study |
| pKH*Ng*Ago-F-*glnR*-*sfGFP*-800 bp::*M. smegmatis* | The strain to knock out *glnR* | This study |
| **Plasmids** |  |  |
| pET28a | Vector with T7 promoter for expression of proteins in *E. coli* BL21(DE3) with His tag | Thermo Scientific |
| pKC1139 | Vector with a temperature-sensitive pSG5 replicon | Bio SCI |
| pET28a-*Ng*Ago-F | Vector with T7 promoter for expression of *Ng*Ago-F in *E. coli* BL21(DE3) with His tag | This study |
| pKH*Ng*Ago-F | pKC1139 with hsp60 promoter-*Ng*Ago-F inserted by [homologous recombination](javascript:;) | This study |
| pKH*Ng*Ago-F-*glnR*-*sfGFP*-100 bp | pKH*Ng*Ago-F with *glnR* homology arms and *sfGFP* inserted by [homologous recombination](javascript:;) | This study |
| pKH*Ng*Ago-F-*glnR*-*sfGFP*-200 bp | pKH*Ng*Ago-F with *glnR* homology arms and *sfGFP* inserted by [homologous recombination](javascript:;) | This study |
| pKH*Ng*Ago-F-*glnR*-*sfGFP*-400 bp | pKH*Ng*Ago-F with *glnR* homology arms and *sfGFP* inserted by [homologous recombination](javascript:;) | This study |
| pKH*Ng*Ago-F-*glnR*-*sfGFP*-600 bp | pKH*Ng*Ago-F with *glnR* homology arms and *sfGFP* inserted by [homologous recombination](javascript:;) | This study |
| pKH*Ng*Ago-F-*glnR*-*sfGFP*-800 bp | pKH*Ng*Ago-F with *glnR* homology arms and *sfGFP* inserted by [homologous recombination](javascript:;) | This study |
| pKH*Ng*Ago-F-*ltmA*--*sfGFP* | pKH*Ng*Ago-F with *ltmA* homology arms and *sfGFP* inserted by [homologous recombination](javascript:;) | This study |

**Table S2 The oligonucleotides used in the study**

| Oligonucleotides | Sequence (5’to 3’) |
| --- | --- |
| **Primers for overproduction of *Ng*Ago (F) protein** | |
| 28a-*Ng*Ago-F-F | CACGCGATGCCGGGGGACGCGGACA |
| 28a-*Ng*Ago-F-R | CAGGAAGCCGACGTTGGACTCGAAG |
| pET28a-F | CTTCGAGTCCAACGTCGGCTTCCTGAAGCTTGCGGCCGCACTCGA |
| pET28a-R | TGTCCGCGTCCCCCGGCATCGCGTGGAATTCGGATCCGCGACCCA |
| T7 | TAATACGACTCACTATAGGG |
| T7 Ter | TGCTAGTTATTGCTCAGCGG |
| **Primers for construction of** **pKH*****Ng*Ago-F** | |
| Hsp60-F | GCCAGTGCCAAGCTTAAGCTTTCTAGAGG |
| Hsp60-F1 | AAGCTTTCTAGAGGATCCGCGGCCGCGCGCGATATCGGTGACCACAACGACGCGCC |
| Hsp60-R | TGCGAAGTGATTCCTCCGGATCGGG |
| Flag-*Ng*Ago-F | CGAATTGGCGCGCGGAATTCTCACAGGAAGCCGACGTTGGACT |
| Flag-*Ng*Ago-R | CCCGATCCGGAGGAATCACTTCGCAGGTACCATGGACTACAAGGA |
| pKC1139-F | AAGCTTGGCACTGGCCGTCGTTTTA |
| pKC1139-R | TGAGAATTCCGCGCGCCAATTCG |
| cx-*Ng*Ago-R | CGCGCTCCCGATTCCGGAAG |
| cx-*Ng*Ago-F | ACGACCGAGCGCAGCGAGTC |
| **Primers for construction of** **pKH*Ng*Ago-F-*glnR*-*sfGFP*** | |
| *glnR-*UHA-F | CGACGGCCAGTGCCAAGCTTTCGTCGGCGGTGTTGGGGCC |
| *glnR-*UHA-R | TCTAGAGTCCTCCCGGCTCGTCAGGA |
| hsp60-GFP-F | CCTGACGAGCCGGGAGGACTCTAGAGGTGACCACAACGACGCGCC |
| UHA-hsp60-gfp-R | TGCGAAGTGATTCCTCCGGATCGGG |
| hsp60-GFP-F2 | CCCGATCCGGAGGAATCACTTCGCAGTGAGCAAGGGCGAGGAGCT |
| *glnR*-GFP-R | GGTGCGCCACTCGGTGGAGGGAATTCTTACTTGTACAGCTCGTCCA |
| *glnR*-DHA-F | GAATTCCCTCCACCGAGTGGCGCACC |
| *glnR*-DHA-R | GCGGATCCTCTAGAAAGCTTCTCGACGTACAGCAGCACCG |
| UHA600-F | TCGCGCGCGGCCGCGGATCCTCTAGAAAGCTTGCGTGAGGGCGCCTCGAC |
| DHA600-R | TAAAACGACGGCCAGTGCCAAGCTTCCTGGAAGAGGCCCTCGGG |
| UHA400-F | TCGCGCGCGGCCGCGGATCCTCTAGAAAGCTTTGGCCCGGATCTCGATCTCG |
| DHA400-R | TAAAACGACGGCCAGTGCCAAGCTTCGGCGCATCTGCAGCAGC |
| UHA200-F | TCGCGCGCGGCCGCGGATCCTCTAGAAAGCTTCCGACCACCAGTCTGCG |
| DHA200-R | TAAAACGACGGCCAGTGCCAAGCTTCTCGGCAGGCGCGAGG |
| UHA100-F | TCGCGCGCGGCCGCGGATCCTCTAGAAAGCTTCAAAGGCCGGTAACGGCTACATGT |
| DHA100-R | TAAAACGACGGCCAGTGCCAAGCTTCCGACCGGCGCGACACC |
| pKH*Ng*Ago-F-F | AAGCTTTCTAGAGGATCCGCGGCC |
| pKH*Ng*Ago-F-R | AAGCTTGGCACTGGCCGTCGTTTTAC |
| *glnr*-F | GTCTTCCTGAACCCCGCCAG |
| *glnr*-R | GGTGCGCCACTCGGTGGAGG |
| **Primers for construction of** **pKH*Ng*Ago-F-*ltmA*-*sfGFP*** | |
| *lmtA*-DHA-F | GTAAAACGACGGCCAGTGCCAAGCTTGACCGAGAAGGCCAGACCCT |
| *lmtA*-DHA-R | GAATTCACTCGCGGTGAGGA |
| *GFP*-R | TCCTCACCGCGAGTGAATTCTTACTTGTACAGCTCGTCCA |
| UHA-hsp60-GFP-R | AGCGTCCGGACACTCTAGAGGTGACCACAACGACGCGCC |
| *lmtA*-UHA-F | TCTAGAGTGTCCGGACGCTAC |
| *lmtA*-UHA-R | GCGGATCCTCTAGAAAGCTTCGGGATCTTCCGCGGCGA |
| *lmtA*-F | ACGAACAGTTCGCGGCCATG |
| *lmtA*-R | TCGAGGGCGAACACCGATCC |
| **Primers for construction of** **qPCR** | |
| *glnR*-qPCR-F | CTCGATGTCACCCTGGCC |
| *glnR*-qPCR-R | GGTTGACGCCTCGATGATCT |
| *ltmA*-qPCR-F | CACTGTCGAACGTCTGGGAC |
| *ltmA*-qPCR-R | AATAGGTGTAGGCGGTTGCC |
| *sigA-*qPCR-F | TGGTGTAGCGGTGGAATG |
| *sigA*-qPCR-R | CGTTTACGGCGTGGACTA |

>ATGCACGCGATGCCGGGGGACGCGGACATGTTCATCGGCATCGACGTGTCGCGCTCGTACCCGGAGGACGGGGCGTCGGGCCAGATCAACATCGCGGCGACCGCGACCGCCGTGTACAAGGACGGGACGATCCTGGGCCACTCGTCGACCCGCCCGCAGCTGGGCGAGAAGCTGCAGTCGACCGACGTGCGCGACATCATGAAGAACGCGATCCTGGGCTACCAGCAGGTGACGGGGGAGAGCCCGACCCACATCGTGATCCACCGCGACGGCTTCATGAACGAGGACCTGGACCCCGCCACCGAGTTCCTGAACGAGCAGGGCGTGGAGTACGACATCGTCGAGATCCGCAAGCAGCCGCAGACCCGCCTGCTGGCGGTCTCGGACGTGCAGTACGACACCCCGGTGAAGTCCATCGCCGCGATCAACCAGAACGAGCCGCGCGCGACCGTGGCCACCTTCGGCGCGCCGGAGTACCTCGCGACCCGCGACGGCGGCGGCCTGCCGCGCCCGATCCAGATCGAGCGCGTCGCGGGCGAGACCGACATCGAGACGCTGACCCGCCAGGTGTACCTGCTGTCGCAGAGCCACATCCAGGTGCACAACAGCACCGCGCGGCTCCCGATCACCACCGCGTACGCGGACCAGGCGTCGACCCACGCGACCAAGGGCTACCTGGTGCAGACCGGCGCCTTCGAGTCCAACGTCGGCTTCCTG

**Figure S1 Optimized *Ng*Ago-F sequence.** Optimization of the *Ng*Ago-F sequence for using as a more suitable base editing tool in *M. smegmatis*.

Mutations of Genomic Analysis and Sequencing (KO *glnR* upstream-Hsp60-GFP-KO *glnR* downstream):

>TCGTCGGCGGTGTTGGGGCCCGTGAGGATGCCGGTCGCCGTCAGCACCAGCGTGCTCTGGTCGGGTCCCTCGAGCGAAAGGTCGACGGCGACGCTGACCCTCTTGTCCAGGCCCGACGCCTTGGGTGTGCCGGTGAAAACCAGACCCGTATTACCGGAGATGCCGGATTCGGTGGTGCCGCCGGTGGCGTCGTTGGTGTCGCGTGAGGGCGCCTCGACCAGCAGGTCGTCGATGCCCATGTAGCGGCCGACATGCGTGGAATCGATGATGATGCGGCTCTCGGCCTCGGCGACCGGGAGCGGTGCGTCCTCGCGGATCAGCCACGAGGCCTCGGTGATGTCGACGTCGTGCAGTGTCGCCTCCAGCGACACCTTTTCGGCGACGGGATGGTCGACGCCGTTGGCCCGGATCTCGATCTCGTTGTATCTGTGGTCGCGGGCCTGCGGGATGAACGGGAACCCGAGGATCGCGACCCACGGATCCCAGTTGAGCGACGCGGCGCTGCGGACCGTACGGGCCAGCCGGTACTCGGCGTAGATGGCTGCTCCGAAATCGGTGCCGACCGCCCCGACGACGAGCGCGGCCACCGCCGCGACGAACCCGACCACCAGTCTGCGCACCGGGACATTGTTGCCCACGAGACCGGCAAAGATTCGCTCGGCGCGGCTGACGAGCAGGTGAGGCGTTATCCTCGTAACACCAAAGGCCGGTAACGGCTACATGTCAGTCATGAATCCGGAAGATCACCCGACACCGGCGTCTGTGTTCTCCCGCAGAGATTCCTGACGAGCCGGGAGGACtctagaGGTGACCACAACGACGCGCCCGCTTTGATCGGGGACGTCTGCGGCCGACCATTTACGGGTCTTGTTGTCGTTGGCGGTCATGGGCCGAACATACTCACCCGGATCGGAGGGCCGAGGACAAGGTCGAACGAGGGGCATGACCCGGTGCGGGGCTTCTTGCACTCGGCATAGGCGAGTGCTAAGAATAACGTTGGCACTCGCGACCGGTGAGTCGTAGGTCGGGACGGTGAGGCCAGGCCCGTCGTCGCAGCGAGTGGCAGCGAGGACAACTTGAGCCGTCCGTCGCGGGCACTGCGCCCGGCCAGCGTAAGTAGCGGGGTTGCCGTCACCCGGTGACCCCCGGTTTCATCCCCGATCCGGAGGAATCACTTCGCAgtgagcaagggcgaggagctgttcaccggggtggtgcccatcctggtcgagctggacggcgacgtaaacggccacaagttcagcgtgtccggcgagggcgagggcgatgccacctacggcaagctgaccctgaagttcatctgcaccaccggcaagctgcccgtgccctggcccaccctcgtgaccaccctgacctacggcgtgcagtgcttcagccgctaccccgaccacatgaagcagcacgacttcttcaagtccgccatgcccgaaggctacgtccaggagcgcacaatcttcttcaaggacgacggcaactacaagacccgcgccgaggtgaagttcgagggcgacaccctggtgaaccgcatcgagctgaagggcatcgacttcaaggaggacggcaacatcctggggcacaagctggagtacaactacaacagccacaacgtctatatcatggccgacaagcagaagaacggcatcaaggtgaacttcaagatccgccacaacatcgaggacggcagcgtgcagctcgccgaccactaccagcagaacacccccatcggcgacggccccgtgctgctgcccgacaaccactacctgagcacccagtccgccctgagcaaagaccccaacgagaagcgcgatcacatggtcctgctggagttcgtgaccgccgccgggatcactctcggcatggacgagctgtacaagtaagaattcCCTCCACCGAGTGGCGCACCGGGCTCACGGGTGCCCAGCAGGCAGAGATTCGCGCGCTGATCGACGCGGCCACCACGCACGACGGTGTCGCGCCGGTCGGTGACCAAGTGCTGCGGGAACTGGGACGCGACCGCACCCGGCACCTGCTGACCACCGACGACGACCGCGTGGTCGGATACCTCAACCTCGCGCCTGCCGAGGGGGACGATCCGGCGATGGCCGAACTCGTCGTGCATCCGCAGGCCCGCCGGCGCGGTATCGGTGCGGCCATGGCGCGCACCGCGCTGGCAGAGGGCGGGCCGGGCGCCCGTATCTGGGCGCACGGCAACATCGCCGCCGCCCAGGCGATGGCGTCATCGCTTCGCCTGGTGGTGGTGCGTGAGCTGCTGCAGATGCGCCGCCCCCTGACCGATCTGCCGCCGGTGCCGGACACCCCCGGCGTGCGCATCGCGACCTACGCCGGCCCCGGCGACGACGCCGAGATCCTGCGGGTCAACAACGCCGCGTTCTCGTGGCACCCCGAGCAGGGCGGCTGGACCGAACACGAGATCGACGAGCGCCGCAACGAGGGGTGGTTCGACCCCGAGGGCCTCTTCCAGGCGTTCGACGAGCAGACCGGCTCGCTCCTGGGATTCCACTGGACCAAGATCCACGACGCGTCCCTCGGCGAGGTCTACGTCGTCGGCGTCGACCCGCAGGCGCAGGGGCGTGGCCTCGGTTACACCCTCACCCTCATCGGCCTGCACCATCTGGCGGAAAAGCTCGCGGGGCCCGAGCCCACGGTGCTGCTGTACGTCGAG

**Figure S2 Mutations of genomic analysis and sequencing when *glnR* were knock out.** Dark blue represents the UHA of *glnR*. Light blue represents the DHA of *glnR*. Dark green represents the *hsp*60 promoter. Light green represents the *GFP*. Black represents two restriction sites. tctaga is *Xba* I and gaattc is *EcoR* I.

Mutations of Genomic Analysis and Sequencing (KO *ltmA* Upstream-Hsp60-GFP-KO *ltmA* Downstream):

>CGGGATCTTCCGCGGCGAGGGCCTGCCCCGTGGTCTGGAAGGCGAATTCGACTGCGAGTGCGTGGTCACCGACCGCTAGTCGAGCGTGCCGGCCTCTCCCGAGATCCGTTCCAGCACGCGCATCGAACCGGTCGCGGACACCTCACGGAACGCTCCGGTGGCCAGCGCGCGCTGGTAGATGTGGAACGGTGCCTGACCGCCGTCGTCGGGATTCGGGAACACGTCGTGGATGACCAGGGCGCCGCCCTTCTGCACCCAACGGGCCCAGCCGTCGAAGTCGCGCTGCGCGGCCTCCTCGGTGTGACCGCCGTCGATGAACAGGAACCGCAACGGGGTTCGCCATCCCCTGGCCACCACGGGTGACCTGCCGACGACGGCGACCACGTGATCGTCGAGGCCCGCGGCGTCGAGCGTGTGCCGCATCTTCGGCAGCGTGTCGAACAGACCGGTCACCGGGTCGACCAGCGACGCGTCGTGGTACTCCCAGCCGGGCTGGTGCTCCTCGGAGCCGTGGTGGTGGTCGACGGTGTAGAGCACCGAGCCGGTCTCCTGCGCGGCCGCGCCGAGCATGACCGTGGACTTGCCGCAGTAGGTGCCGATCTCGACGCCGACGCCGCCGCCGAGGTAGCGCACCGCCGCGTCGTAGAGGGCTCGGCCCTCGTCAACGGGCATGAAACCCGTGACCTGCTCGGCCAGGGCGAACAGACGCTCGGCGCGGGGCGGCAGTGCGGTGTCGGCATGGCTCATGGGTGACAACATACCGTTGCTGGTCACGGCCAGTTGTAGTAGCGTCCGGACACtctagaGGTGACCACAACGACGCGCCCGCTTTGATCGGGGACGTCTGCGGCCGACCATTTACGGGTCTTGTTGTCGTTGGCGGTCATGGGCCGAACATACTCACCCGGATCGGAGGGCCGAGGACAAGGTCGAACGAGGGGCATGACCCGGTGCGGGGCTTCTTGCACTCGGCATAGGCGAGTGCTAAGAATAACGTTGGCACTCGCGACCGGTGAGTCGTAGGTCGGGACGGTGAGGCCAGGCCCGTCGTCGCAGCGAGTGGCAGCGAGGACAACTTGAGCCGTCCGTCGCGGGCACTGCGCCCGGCCAGCGTAAGTAGCGGGGTTGCCGTCACCCGGTGACCCCCGGTTTCATCCCCGATCCGGAGGAATCACTTCGCAgtgagcaagggcgaggagctgttcaccggggtggtgcccatcctggtcgagctggacggcgacgtaaacggccacaagttcagcgtgtccggcgagggcgagggcgatgccacctacggcaagctgaccctgaagttcatctgcaccaccggcaagctgcccgtgccctggcccaccctcgtgaccaccctgacctacggcgtgcagtgcttcagccgctaccccgaccacatgaagcagcacgacttcttcaagtccgccatgcccgaaggctacgtccaggagcgcacaatcttcttcaaggacgacggcaactacaagacccgcgccgaggtgaagttcgagggcgacaccctggtgaaccgcatcgagctgaagggcatcgacttcaaggaggacggcaacatcctggggcacaagctggagtacaactacaacagccacaacgtctatatcatggccgacaagcagaagaacggcatcaaggtgaacttcaagatccgccacaacatcgaggacggcagcgtgcagctcgccgaccactaccagcagaacacccccatcggcgacggccccgtgctgctgcccgacaaccactacctgagcacccagtccgccctgagcaaagaccccaacgagaagcgcgatcacatggtcctgctggagttcgtgaccgccgccgggatcactctcggcatggacgagctgtacaagtaagaattcACTCGCGGTGAGGATCGCCCGCGACGCCGCGACGGCCGGTTCGTAGATCTCCCGGATGTCGTGGCCGTCCTCGACGATCAGTGCCGTGAGTTCCCGTAGACCGCCCAGCAGGATGATGGCCATCTGCCTGCTGAGCCCGTTGAGCCCGGCGCGCCGGAATCCCGGACTGTCGCTGAGGTCGATGAGCAGATCGGTGAGCGCCCCCATCGCGTCGCGTTGCAGCGGGCGTGCCGCGGGGCCGAGCGCGGGCAGTTCGCGGATCCAGCTCAGCGTGATGCTCGGGGCGGATTCGATGTGCTCGACGTAGGCGCCGACGGCCTGGTCGATCTGGTCCTGCCATTCGGCCTCGGGGCTGACGGCCGAGCGGATGTGCTCGACGAGCGCGGCGTTGTTGGCGCGCAGCAGCTCGATGAAACAGGCTTCTTTGCTTGCGAACTGGTCGTAGAACGTGCGCTTGGAGGTGCGAGCGTGGCGGACGATGTCGGCAACGGTGCTGTCGCGGTAACCCTTGTCGTCGATGGCGGTCGAGAGCCCGTCGAGCAGGCGGGACCGGAACGCGTCACCGCCTGAGCCCCCCGCCGTGGCGGTGCCGGTGAGCGCGGTCGTCATACGGACCCCCCTTGCGCTGTGTGGTACCTGCGAGTACCGTACTACACCATCACCTGGTACGACGCGGTACCAACAACAGGGGAGAGCCTATGACCGCGACCGTCGAACGTCCCGAGTCCGCGATCCGCGCGGCGCAGCTTCCGCCGGTGCTGCCGCTGCCCAAAACTGTTCAGGGTCTGGCCTTCTCGGTC

**Figure S3 Mutations of genomic analysis and sequencing when *ltmA* were knock out.** Dark blue represents the UHA of *ltmA*. Light blue represents the DHA of *ltmA*. Dark green represents the *hsp*60 promoter. Light green represents the *GFP*. Black represents two restriction sites. tctaga is *Xba* I and gaattc is *EcoR* I.
